# High-Temperature Sensing Based on GAWBS In Silica Single-Mode Fiber

**DOI:** 10.3390/s23031277

**Published:** 2023-01-22

**Authors:** Shaonian Ma, Yuxi Pang, Qiang Ji, Xian Zhao, Yongfu Li, Zengguang Qin, Zhaojun Liu, Yanping Xu

**Affiliations:** 1 Center for Optics Research and Engineering, Shandong University, Qingdao 266237, China; 2 Key Laboratory of Laser and Infrared System of Ministry of Education, Shandong University, Qingdao 266237, China; 3 School of Information Science and Engineering, Shandong University, Qingdao 266237, China

**Keywords:** optical fiber sensors, high-temperature measurement, guided acoustic wave Brillouin scattering, forward Brillouin scattering

## Abstract

High temperature detection is a constant challenge for condition monitoring under harsh environments in optical fiber sensors research. In this study, the temperature response characteristics of guided acoustic wave Brillouin scattering (GAWBS) spectra in silica single-mode fiber (SMF) up to 800 °C are experimentally investigated, demonstrating the feasibility of the method for high-temperature monitoring. With increasing temperature, the resonance frequency of GAWBS spectra increases in a nearly linear manner, with linearly fitted temperature-dependent frequency shift coefficients of 8.19 kHz/°C for *TR*_2,7_ mode and 16.74 kHz/°C for *R*_0,4_ mode. More importantly, the linewidth of the GAWBS spectra is observed to narrow down with increasing temperature with a linearly fitted rate of −6.91 × 10^−4^/°C for *TR*_2,7_ modes and −8.56 × 10^−4^/°C for *R*_0,4_ modes. The signal-to-noise ratio of the GAWBS spectra induced by both modes increase by more than 3 dB when the temperature rises from 22 °C to 800 °C, which indicates that the proposed sensing scheme has better performance in high-temperature environments, and are particularly suitable for sensing applications in extreme environments. This study confirms the potential of high-temperature sensing using only GAWBS in silica fibers without any complex micromachining process, which has the advantages of strong mechanical strength, simple structure, easy operation, and low cost.

## 1. Introduction

Optical fiber high temperature sensing is a constant challenge for condition monitoring in harsh environments, such as aerospace, nuclear energy production, metallurgy, and deep underground wells [1,2,3]. Typically, fiber Bragg gratings (FBGs), [4,5] based on femtosecond (fs) laser inscription, and various interferometric sensors [6,7,8], based on high-precision microcavities, are widely studied, and they have good high temperature measurement performance. FBGs, usually etched in silica SMF by laser, can be operated under high temperature with temperature sensitivity of about 10 pm/°C [9]. Interferometer-based high temperature sensors are usually realized by fabricating micro-cavity structures using laser writing, mode field mismatch by mismatch fusion splicing, fiber tapers by electric arc discharge or oxyhydrogen flame, and so on. According to the different interference structures, high-temperature fiber-optic sensors can be divided into several types, including transmission type, such as Mach-Zehnder interferometers (MZIs), and reflection type, such as Michelson interferometer (MIs), and Fabry–Perot interferometers (FPIs) [10,11]. Most interferometric high temperature fiber-optic sensors can operate with higher temperature sensitivity (10–165 pm/°C) compared with FBG sensors thanks to the higher sensitivity of the optical phase difference (OPD) on temperature variations [10]. Unfortunately, the use of laser inscriptions or the precision fabrication of microstructures unavoidably increases the complexity of sensor preparation and cost, and reduces the mechanical strength of the sensors.

Different from FBGs and interferometers that require precision laser inscription and microstructure fabrication, Brillouin scattering-based fiber-optic sensors utilizing communication SMF without any modifications can also function as high-temperature sensors, by exploiting the relationship between the Brillouin frequency shift (BFS) and temperature [12,13]. According to the propagating direction of the scattered light relative to that of the incident pump light, Brillouin scattering can be divided into backward Brillouin scattering (BBS) when the scattered light counter-propagates against the incident pump light, and forward Brillouin scattering (FBS) when the scattered light propagates along the incident pump light. As a typical acousto-optic interaction, which is easier to be observed in optical fibers, the study of BBS starts earlier and sensors based on BBS are more mature than those based on FBS, due to the larger gain coefficient for BBS in optical fibers [14,15]. In recent years, distributed fiber-optic temperature and/or strain sensing based on BBS has been intensively investigated and is promising for engineering applications [16]. However, to deal with the large BFS (*v*_BBS_ ≈ 10.8 GHz in standard SMF) in BBS-based sensors, costly broadband signal processing modules are required, which increases the cost and complexity of the demodulation system [17]. Moreover, the published results also show that when the temperature increases, the temperature-dependent BFS coefficient changes from 1.349 MHz/°C at 22 °C to 0.419 MHz/°C at 1000 °C [18], which reveals a nonlinear relationship between temperature and BFS. These drawbacks limit the development of BBS-based point high temperature sensors. In contrast, the BFS of FBS in standard SMF is usually in the order of hundreds of MHz, which reduces the requirements of broadband detection devices. In addition, the BFS of FBS is theoretically predicted to vary more linearly with the temperature [19,20], offering great potential for high temperature sensing.

FBS is also called guided acoustic wave Brillouin scattering (GAWBS), in which forward scattering of incident pump wave occurs caused by a series of transverse acoustic modes in the circular cross section of fibers, leading to multi-peak feature of the GAWBS spectrum [21]. The resonance frequency and linewidth of the GAWBS spectrum are determined by the transversely transmitted resonant acoustic waves, which are highly sensitive to fiber diameter, temperature, and acoustic impedance outside fiber cladding region [22,23,24]. Therefore, GAWBS has been widely studied in temperature and strain sensing [19,24], fiber diameter uniformity testing [25], material characterization [26,27], and acoustic impedance measurement of liquids [28,29], even towards simultaneous measurement of multiple parameters [30]. To date, the temperature dependence of the GAWBS frequency shift has been investigated for various types of fibers, including single-mode fibers [19], cavity-assisted fibers [31], photonic crystal fibers [32], highly nonlinear fibers [33], and polyimide coated fibers [23]. However, most of the studies are focused on the temperature-dependent resonance frequency shift coefficient over the low temperature range of −20 to 100 °C, to our knowledge. The spectral characteristics of GAWBS at higher temperatures have not been fully explored. As high temperatures would place impacts on acoustic properties of the silica fiber that affect the spectral characteristics of GAWBS [34,35], it is necessary to investigate the feasibility of GAWBS-based fiber optic sensors for high temperature measurement by experimental investigations.

In this paper, we characterize a standard silica SMF for high-temperature measurement based on polarized and depolarized GAWBS. The resonance frequency of *R*_0,4_ mode and *TR*_2,7_ mode induced GAWBS spectra increases nearly linearly with temperature and has a temperature-dependent resonance frequency shift coefficient of 16.74 kHz/°C and 8.19 kHz/°C, respectively. In the meantime, as the temperature increases, the linewidth of the spectrum becomes narrower and the signal-to-noise ratio (SNR) increases, which originates mainly from the increase in viscose lifetime of acoustic phonons with temperature. Theoretical analysis explaining the phenomenon of increasing SNR with temperature is conducted, in which the simulated result agrees well with the experimental result.

## 2. Theory and Sensing Principle

Standard silica SMF is usually considered as isotropic cylinder due to the similar mechanical properties of the core and cladding materials. In such a waveguide, where GAWBS occurs, the stimulated resonant acoustic waves can be mainly divided into two categories: radial modes (*R*_0,*m*_) and torsional-radial modes (*TR*_2,*m*_); where *m* denotes the order of the acoustic resonance [21]. *R*_0,*m*_ modes perturb the refractive index of the fiber cross-section, thereby modulating the phase of the transmitted light to induce polarized GAWBS, while the *TR*_2,*m*_ modes perturb both the refractive index, and the birefringence to induce depolarized GAWBS. Different from the BBS process which typically produces single Stokes light at a frequency shift of 10.8 GHz, the GAWBS process can produce multiple Stokes lights within a frequency range of 20–800 MHz in silica SMF [21]. The study of the vibrational modes in a uniform cylinder shows that the resonance frequency (GAWBS-induced frequency shift) of the *m*th acoustic mode depends on the cylinder diameter *d* and the acoustic velocity *V*.

(1) $$v_{m}=\frac{Vy_{m}}{\pi d}$$

where *d* is the fiber cladding diameter, which is 125 μm in our case; *v*_m_ corresponds to the resonance frequency of *R*_0,*m*_ mode when *V* is the longitudinal acoustic velocity *V_L_*; *v*_m_ corresponds to the resonance frequency of *TR*_2,*m*_ mode when *V* is the shear acoustic velocity *V_S_*; and *y_m_* is the *m*th solution to the boundary condition equations comprised of Bessel functions [21]:

(2) $$\;\left(1-\alpha^{2}\right)J_{0}\left(y_{m}\right)-\alpha^{2}J_{2}\left(y_{m}\right)=0\;(R_{0,m}\;\mathrm{modes})$$

(3) $$\left|\begin{array}{cc} \left(3-\frac{y_{m}^{2}}{2}\right)J_{2}\left(\alpha y_{m}\right) & \left(6-\frac{y_{m}^{2}}{2}\right)J_{2}\left(y_{m}\right)-3y_{m}J_{3}\left(y_{m}\right)\\ J_{2}\left(\alpha y_{m}\right)-\alpha y_{m}J_{3}\left(\alpha y_{m}\right) & \left(2-\frac{y_{m}^{2}}{2}\right)J_{2}\left(y_{m}\right)+y_{m}J_{3}\left(y_{m}\right) \end{array}\right|=0\;(TR_{2,m}\;\mathrm{modes})$$

where *J*_0_, *J*_2_, and *J*_3_ are the zero-order, second-order, and third-order Bessel functions, respectively, *α* equals to *V*_S_/*V_L_*. For fused silica, vs. = 3764 m/s, and *V_L_* = 5968 m/s, *α* = 0.63 [36]. *V_L_*, vs., and *α* are dependent on the acoustic properties of the material and their expression are given as follows [19]:

(4) $$\;V_{L}=\sqrt{\frac{E\left(1-\gamma \right)}{\left(1+\gamma \right)\left(1-2\gamma \right)\rho }}$$

(5) $$V_{S}=\sqrt{\frac{E}{2\rho \left(1+\gamma \right)}}$$

(6) $$\alpha =\sqrt{\frac{1-2\gamma }{2\left(1-\gamma \right)}}$$

where *ρ* = 2203 kg/m^3^ is the density of silica, Δ*ρ*/*ρ* = −1.83 × 10^−6^/°C, *γ* = 0.17 is the Poisson’s ratio, Δ*γ*/*γ* = 6.7 × 10^−5^/°C, *E* = 72.553 GPa is the Young’s modulus [37,38]. For the thermal response of GAWBS, the resonance frequencies of polarized GAWBS and depolarized GAWBS depend on the longitudinal acoustic velocity *V_L_* and shear acoustic velocity vs., respectively, and *y_m_*, but independent of the effective refractive index *n* according to Equation (1). In Equations (2) and (3), it is shown that *y_m_* is related to *α*, which equals to *V_S_*/*V_L_*. To evaluate the temperature dependence of these parameters, theoretical calculations are performed, and it is found that the value of *α* changes from 0.63 to 0.617 for a temperature increase of 1000 °C [20], which results in the change of *y_m_* of less than 0.1%. For comparison, the increase in *V_L_* or vs. could be as large as 8% for a temperature increase of 1000 °C. Thus, in Equation (1), *y_m_* can be considered as a fixed value that does not vary with temperature due to its much weaker dependence on temperature compared to the acoustic velocities. Since *π* is a constant, and *d* does not vary significantly with temperature variations in Equation (1), the resonance frequency of each acoustic mode can be finally considered to be approximately linearly dependent on the temperature in the qualitative theoretical prediction.

In addition to the resonance frequencies corresponding to different acoustic modes, linewidth of the resonance peak in the GAWBS spectra is also an important parameter for characterizing the GAWBS spectra. The linewidth of GAWBS spectra in uncoated silica SMF can be expressed as [39]:

(7) $$\Gamma_{m}=\Gamma_{inhomo}+\Gamma_{\tau }$$

where *Γ*_*inhomo*_ = (*δd*/*d*)*v_m_*·× 2π, originated from the variation of diameter *δd*, which is negligible with temperature change [39]. *Γ*_*τ*_ = 1/*τ* is the viscose damping term, which is inversely related to the viscose lifetime *τ*. As the temperature increases, the viscosity of the FUT increases [40,41], which enhances the phonon lifetime and the associated forward Brillouin scattering efficiency, resulting in the linewidth narrowing.

## 3. Experimental Results

### 3.1. Experimental Setup

**Figure 1:**
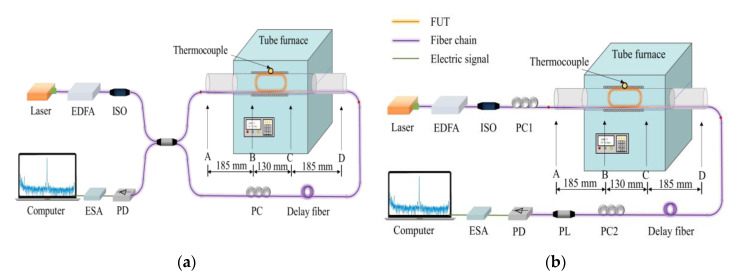
Schematic setup of the high temperature sensing system based on (**a**) polarized GAWBS, and (**b**) depolarized GAWBS. EDFA, erbium-doped fiber amplifier; ISO, isolator; PC, polarization controller; FUT, fiber under test; PL, polarizer; PD, photo detector; ESA, electrical spectrum analyzer.

A standard silica SMF (manufactured by YOFC (Wuhan, China)) with a cladding diameter of 125 μm, a mode field diameter (MFD) of 10.8 μm, and an effective refractive index (*n*_eff_) of 1.467 at 1550 nm, is used as the fiber under test (FUT). The high-temperature heating device is a temperature-controlled tube furnace with an effective heating zone extending over a length of about 13 cm (in Figure 1), and a thermocouple is placed in the center of the quartz tube for temperature monitoring. As shown in Figure 1, the FUT is wound into a coil with diameters that are fitted with the inner diameter of the heating tube as a high temperature sensing element. Before temperature measurement for each new sample, the FUT is first heated to 800 °C, then kept at the temperature for around 1 h to remove the fiber coating through the coating burning effect [42]. The FUTs used in the subsequent experiments are all annealed fibers that have been heated and cooled down to room temperature naturally with fiber coating removed.

The schematic diagram for investigating the temperature response of the polarized and depolarized GAWBS spectrum is illustrated in Figure 1. For both configurations, output light from a narrow linewidth semiconductor laser (NLLD-0175-3-34-2, center wavelength: 1550.12 nm, linewidth: 15 kHz) is amplified to 19 dBm by an erbium-doped fiber amplifier (EDFA) as the pump light. An isolator (ISO) is placed after the EDFA to block reflected light for protection. As shown in Figure 1a, when pump light enters the Sagnac loop consisting of a 50:50 fiber coupler, this interferometer structure converts the acoustic wave-induced phase modulation to intensity modulation. In the above setup, radial and torsional-radial acoustic waves can both be excited [23], so it is necessary to adjust the polarization controller (PC) in the loop to suppress the torsional-radial acoustic modes as much as possible, and retain only the polarized GAWBS spectrum induced by radial acoustic modes. Then, the beat signal of the pump light and the scattered light by polarized GAWBS is detected by a fast photodetector (PD, bandwidth: 350 MHz). Finally, the polarized GAWBS spectrum is observed by an electrical spectrum analyzer (ESA, bandwidth: 6.2 GHz) with a frequency resolution of 1 kHz after being averaged by 1000 times. Unlike the setup shown in Figure 1a, the setup in Figure 1b is used to excite and demodulate depolarized GAWBS process in FUTs two PCs, and a polarizer (PL) are used to convert the *TR*_2,*m*_ modes induced polarization modulation to amplitude modulation. Similar to Figure 1a, the beat signal of the pump light and the scattered light by depolarized GAWBS is detected by a PD, the spectrum of which is analyzed by the ESA.

### 3.2. Results and Discussion

**Figure 2:**
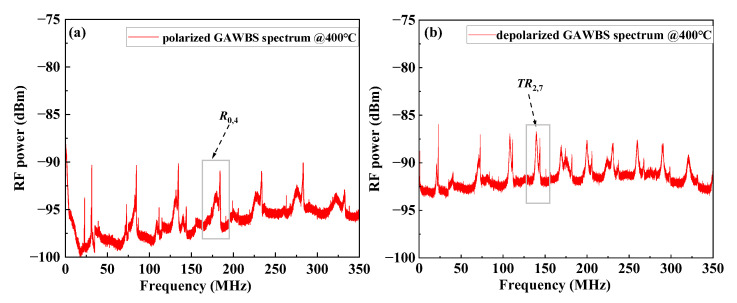
Measured polarized (**a**) and depolarized (**b**) GAWBS spectrum at 400 °C.

Figure 2a,b show the measured polarized and depolarized GAWBS spectra at 400 °C by using the above configurations, respectively. A series of resonance peaks corresponding to the first 7 orders of *R*_0,*m*_ modes (*m* = 1,2,…,7) can be observed in Figure 2a, in which the spectrum is also mixed with low-frequency noise caused by the external environmental disturbances and incompletely suppressed resonance peaks induced by low-order *TR*_2,*m*_ modes (*m* = 1,3,4,7). Although the *TR*_2,*m*_ modes induced depolarized GAWBS resonance peaks can be suppressed by adjusting the PC in the experimental configuration at low temperatures, these resonance peaks reemerge with the increasing temperatures as both polarized and depolarized GAWBS would be enhanced with temperature increase (detailed analysis later). Resonance peaks in Figure 2b correspond to the first 16 orders of *TR*_2,*m*_ modes (*m* = 1,2,…,16), which have higher radio frequency (RF) powers without the use of a 50:50 fiber coupler in the setup shown in Figure 1b. Considering that the resonance peaks corresponding to the *R*_0,4_ mode (near 178 MHz in Figure 2a) and *TR*_2,7_ mode (near 140 MHz in Figure 2b) have a relatively large SNR, their temperature dependence are investigated [21]. The acoustic mode order of the resonance peaks in the GAWBS spectra obtained from the experiments for both polarized and depolarized GAWBS can be identified according to the calculated values of the corresponding resonance frequencies using Equations (1)–(3). The numerically calculated results of the resonance frequencies for both polarized and depolarized GAWBS in silica SMF, and the corresponding measured results are listed in Table 1.

**Figure 3:**
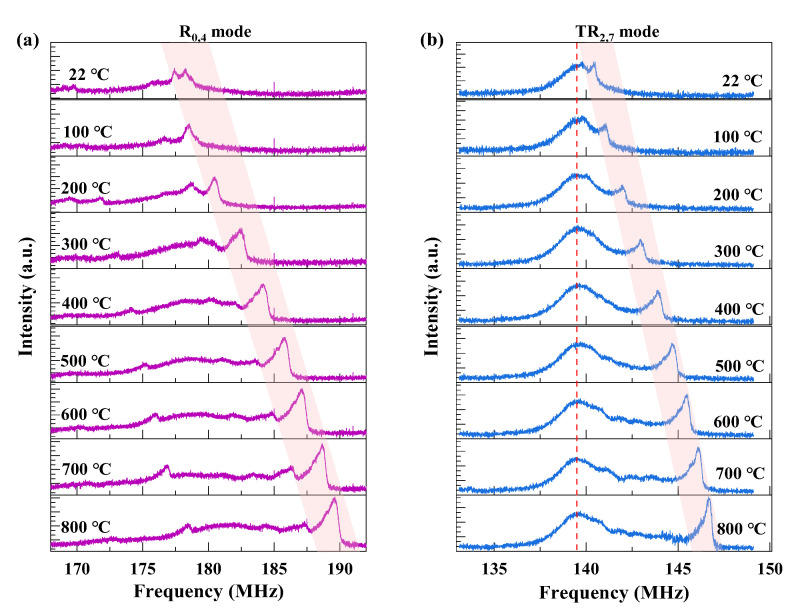
Measured (**a**) *R*_0,4_ mode induced polarized GAWBS spectrum, and (**b**) *TR*_2,7_ mode induced depolarized GAWBS spectra at temperatures ranging from 22 °C to 800 °C.

To investigate the temperature dependence of the GAWBS spectra, the temperature of the furnace tube is changed from 22 °C to 800 °C with a step of 100 °C. Figure 3 shows the test results of the *R*_0,4_ mode and *TR*_2,7_ mode induced spectra at different temperatures. It is clearly seen that the center frequency of the resonance peak increases with increasing temperature. Besides the center frequency, it can be found that the SNR of the GAWBS resonance peak of the FUT also increases with the temperature (detailed analysis later), while the linewidth of the peak narrows down with the temperature. Since the two modes have similar thermal response to temperature, and the *TR*_2,7_ mode induced GAWBS spectra have higher RF power, narrower linewidth, and less noise interference from the intrinsic noise of the instruments, *TR*_2,7_ mode is selected as an example for detailed analysis.

**Figure 4:**
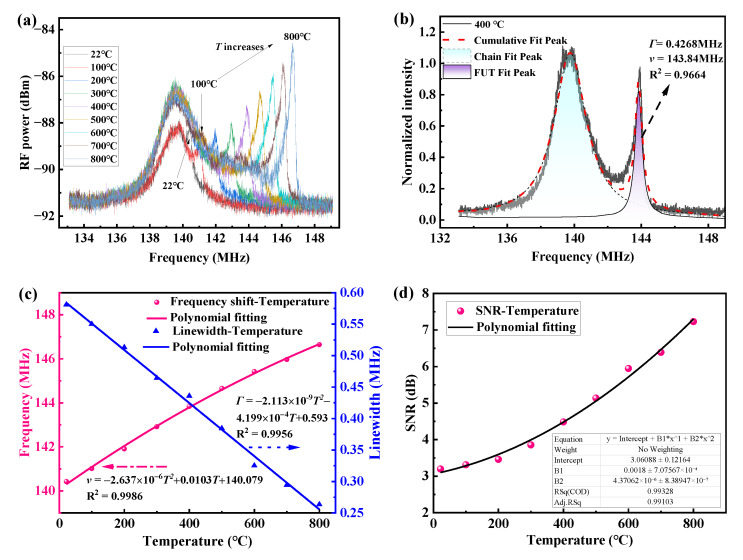
(**a**) Measured *TR*_2,7_ mode induced depolarized GAWBS spectra at temperatures ranging from 22 °C to 800 °C; (**b**) Normalized *TR*_2,7_ mode induced depolarized GAWBS spectrum at 400 °C and the corresponding Lorentzian multi-peak fitting result; (**c**) Resonance frequency and linewidth of *TR*_2,7_ mode induced depolarized GAWBS spectra as a function of temperature; (**d**) Measured SNR of the *TR*_2,7_ mode induced depolarized GAWBS spectra as a function of temperature.

The measured results of the *TR*_2,7_ mode induced GAWBS spectra at different temperatures, normalized and Lorentz fitting spectrum at 400 °C, the temperature-dependent resonance frequency shift and linewidth variation of the resonance spectra, and the temperature-dependent SNR of the resonance spectra are shown in Figure 4. In Figure 4a, it is seen that each spectrum measured at each temperature consists of two peaks, one with an almost fixed lower frequency corresponding to the depolarized GAWBS occurring along the fiber chain including delayed fiber and fiber patch cords, and the other one with higher increasing frequencies when temperature increases corresponding to the depolarized GAWBS occurring along the FUT. The center frequency of the first resonance peak contributed by the fiber chain can be used as a reference at room temperature for the absolute temperature measurement when monitoring the center frequency of the second resonance peak in Figure 4a. Note that as the length of the FUT is smaller than that of the fiber chain, the strength of depolarized GAWBS in the FUT is normally smaller than that in the fiber chain at relatively low temperatures, resulting in a higher SNR value of the first resonance peak than that of the second peak in Figure 4a, as observed for temperatures below 600 °C.

**Figure 5:**
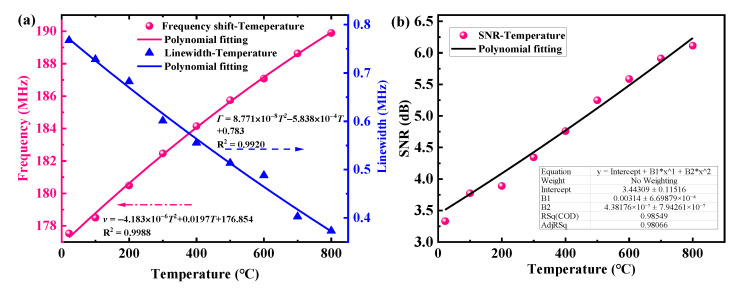
(**a**) Resonance frequency and linewidth of *R*_0,4_ mode induced polarized GAWBS spectra as a function of temperature; (**b**) Measured SNR of the *R*_0,4_ mode induced polarized GAWBS spectra as a function of temperature.

In order to accurately quantify the variation of resonance frequency and linewidth, the spectrum is normalized, and Lorentzian multi-peak fitted as exemplified in Figure 4b, which shows the depolarized GAWBS spectrum of *TR*_2,7_ mode measured at 400 °C. As illustrated in Figure 4b, both of the measured depolarized GAWBS spectral peaks of *TR*_2,7_ mode contributed by the fiber chain at room temperature and the FUT at 400 °C are well fitted with Lorentzian fitting method. The goodness of fitting for the resonance peak of *TR*_2,7_ mode at 400 °C is obtained with a high R^2^ value of 0.9664. In the fitting result, the center frequency of the second resonance peak as induced by the FUT is found to be shifted from 140.42 MHz at room temperature to 143.84 MHz at 400 °C. The linewidth of the *TR*_2,7_ mode spectral peak is measured to be 0.4268 MHz at 400 °C. The same treatment is then applied to the spectra obtained at other temperatures to extract the resonance frequency and the linewidth, which are then mapped as a function of temperature as shown in Figure 4c. Then, Polynomial fitting is applied on the raw data, and the obtained fitted functions are added in Figure 4c. It is found that both the resonance frequency and the linewidth of the depolarized GAWBS spectrum are approximately linearly related to the increasing temperature. In Figure 4c, it can be found that the resonance frequency shifts nearly linearly with increasing temperature at lower temperatures (<400 °C). However, as the temperature continues to increase beyond 400 °C, the frequency growth rate begins to decay. In addition to the polynomial fitting in Figure 4c, linear fitting is also performed on the resonance frequency and the linewidth data, and the obtained R^2^ values are 0.9937 and 0.9715, respectively, which indicates that the variations of the resonance frequency and linewidth of the *TR*_2,7_ mode GAWBS spectrum follow an approximately linear relationship with the temperature over the tested range. The temperature-dependent resonance frequency shift coefficient and linewidth variation coefficient of the *TR*_2,7_ mode is found to be 8.19 kHz/°C and −0.409 kHz/°C, respectively. These linear fitting results are also used in the subsequent theoretical calculation of the temperature dependence of the GAWBS gain coefficient. Thus, with the linearly fitted temperature-dependent resonance frequency shift coefficient and linewidth coefficient, the resonance frequency shift rate (Δ*v*/*v*) is calculated to be (5.89 ± 0.06) × 10^−5^/°C, and the linewidth variation rate (Δ*Γ*/*Γ*) is (−6.91 ± 0.14) × 10^−4^/°C for *TR*_2, 7_ modes. In addition, the same operation is applied to the measurement results of *R*_0,4_ mode induced polarized GAWBS spectra at different temperatures. Similar relationship between frequency shift (or linewidth) and the temperature is obtained as shown in Figure 5. It can be found that the frequency increases nearly linearly with temperature when the temperature is below 400 °C, and the rate of increase begins to decay as the temperature continues to rise. Similarly, both polynomial fitting and linear fitting are performed on the raw data, and the obtained polynomial fitting functions are added in Figure 5. In the linear fitting results, R^2^ values for resonance frequency and linewidth are 0.9950 and 0.9804, respectively. The temperature-dependent resonance frequency shift coefficient and the linewidth variation coefficient are extracted to be 16.74 kHz/°C and −0.512 kHz/°C, respectively. As a result, the frequency shift rate and the linewidth variation rate for *R*_0,4_ mode are calculated to be (9.44 ± 0.06) × 10^−5^/°C and (−6.91 ± 0.24) × 10^−4^/°C, respectively.

The SNR of the resonance peak in both the polarized and depolarized GAWBS spectra is defined as the ratio of the resonance peak power to the floor noise power. By subtracting the noise floor (the spectrum when the FUT is removed from the experimental setup) from the raw data of the FUT GAWBS spectra, a series of more accurate resonance peak data can be obtained [43]. From the new data after noise floor compensation, the SNR for each resonance peak can be easily extracted. As indicated in Figure 4d and Figure 5b, the SNR of the *TR*_2,7_ mode and *R*_0,4_ mode induced GAWBS spectrum increases with the temperature. The underlying physics for this relationship can be attributed to the enhancement in the gain coefficient *g* that arises from the enhancement in the coupling efficiency between the optical photons and acoustic phonons in the GAWBS process with temperature increase. In general, a standard GAWBS spectrum g(*Ω*) can be expressed as follows [36]:

(8) $$g\left(\Omega \right)=g\frac{{\left(\Gamma_{m}/2\right)}^{2}}{{\left(\Omega -\Omega_{a}\right)}^{2}+{\left(\Gamma_{m}/2\right)}^{2}}\;g=\frac{\omega_{1}\gamma_{e}^{2}\left|Q_{0}Q_{1}\right|}{2n_{\mathrm{eff}}^{2}c^{2}\rho_{0}\Omega_{a}\Gamma_{m}}$$

where *Ω*_a_ = 2π × *v_m_* is the angle frequency of resonance peak, *ω*_1_ is the pump light angle frequency, *γ*_e_ is the electrostrictive constant, *n*_eff_ is the effective refractive index of silica SMF, *c* is the velocity of light in vacuum, *ρ*_0_ is the mean density of silica. *Q*_0_ and *Q*_1_ are the acoustic-optical coupling factor, which can be calculated numerically by resolving the normalized density variation distribution *ρ*_2,*m*_ of different modes and normalized optical filed distribution *E*_0_ [25]. The gain factor *g*_0_ for the *TR*_2,7_ mode is estimated to be 4.4 × 10^−3^ m^−1^W^−1^, using the initial values at 22 °C: *γ*_e_ = 1.17, *n*_eff_ = 1.467, *ρ*_0_ = 2203 kg·m^−3^, and the experimental values of *ω*_1_ = 2π × 194 THz, *Ω*_a_ = 2π × 140.42 MHz, *Γ_m_* = 2π × 0.59 MHz.

**Figure 6:**
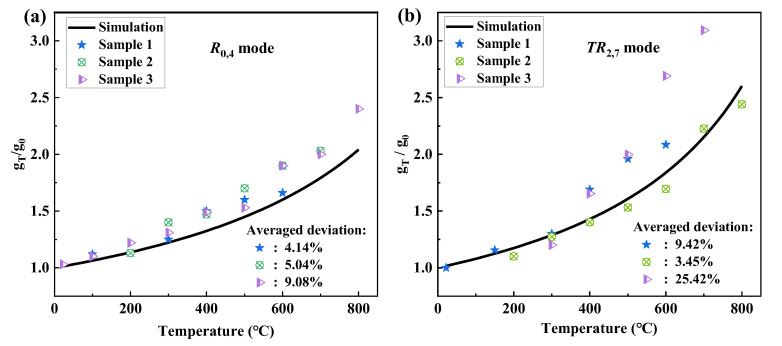
Measured and calculated g_T_/g_0_ of (**a**) *R*_0,4_ mode, and (**b**) *TR*_2,7_ mode induced GAWBS at different temperatures.

When the temperature changes, *Ω*_a_ and *Γ_m_* perform very important roles in the spectral shape and the value of *g_T_* (*T* denotes temperature), *n*_eff_ and *ρ*_0_ also have a weak impact on *g_T_*. The rates of change of the parameters for silica fibers at high temperatures are given by [37]: (Δ*n*_eff_/*n*_eff_) = 7.6 × 10^−6^/°C, (Δ*ρ*_0_/*ρ*_0_) = −1.83 × 10^−6^/°C. As the linewidth *Γ_m_* has the largest rate of change with negative values when temperature increases, the gain coefficient *g_T_* will increase with temperature according to Equation (8). The rate of change obtained in Figure 4c and Figure 5a can be taken into Equation (8) to derive the relationship between the gain coefficient *g_T_* and the temperature. In studying the effect of temperature on the gain coefficient of GAWBS, we selected samples of different lengths for multiple tests to investigate whether the sensing fiber length would affect the high temperature sensing performance. In most of the previously published works on the sensing characteristics of GAWBS in SMF, the FUT lengths chosen by researchers are between 10–15 m [29,30,43], which ensures a relatively highly efficient GAWBS process with high-SNR resonances and at the same time a minimized fiber probe for point sensing. For the above reasons, two groups of FUTs, each consists of three different fiber segments (Sample 1, Sample 2, and Sample 3) with lengths of 10 m, 12 m, and 15 m, respectively, are tested to obtain the gain coefficients *g_T_* at different temperatures in the polarized and depolarized GAWBS experiments. Figure 6 shows the measured and calculated *g_T_*/*g*_0_ of *R*_0,4_ mode and *TR*_2,7_ mode, where *g*_0_ is the gain coefficient at 22 °C. It can be found in Figure 6 that the test results for Sample 1 and Sample 2 for both the *R*_0,4_ and *TR*_2,7_ modes are almost consistent with the simulation results, with the averaged deviation of the experimental test results from the simulated values less than 10%. These deviations mainly come from different tensile strain, bending stress, and torsional deformation distributed along the FUTs when they are manually looped into ring structures and different heat distributions when placed into the furnace tube with narrow space. It is also seen that the test results for Sample 3 for *TR*_2,7_ mode have larger deviations from the simulation results than those for *R*_0,4_ mode, which is mainly due to the fact that the depolarized GAWBS process is more sensitive to the stress/strain-induced birefringence changes along the sensing fiber than the polarized GAWBS as Sample 3 is the longest sample which suffers the most nonuniform stress/strain distributions when looped into the ring structure. Additionally, different tensile strain, bending stress, and torsional deformation, and nonuniform heat distribution introduced to the samples for both mode measurements during the processes of manual looping and insertion into the heat tube in each time would also contribute to the deviation differences. It is believed that the inconsistencies in the current results would be alleviated if the test samples are looped to rings with the same size and placed within high-temperature furnace with enough large space.

**Figure 7:**
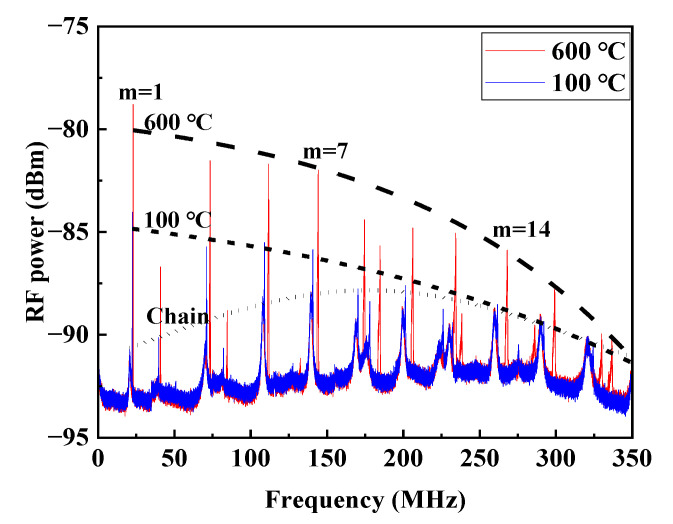
Measured depolarized GAWBS spectra at 100 °C and 600 °C, respectively.

To illustrate the variation of SNR with temperature for all the measured modes of 16 orders, the test results of the 12 m FUT are shown in Figure 7. The peaks of these modes at 100 °C and 600 °C are extracted, and envelope curves covering resonance peaks for all the modes are plotted, respectively. It can be found that the SNR increases with temperature for all the first 15 orders of *TR*_2,*m*_ modes (*m* = 1,2,…, 15), and it almost does not change with temperature for *TR*_2,*m*_ modes of orders *m* > 15. This is because the linewidth *Γ_m_* of the resonance spectrum induced by the depolarized GAWBS in the uncoated fiber increases linearly with *m*, as shown in Equation (7), and the corresponding linewidth changes *δΓ_m_* (100 °C to 600 °C) are different for different acoustic modes when the temperature changes. Moreover, the relationship between *Γ_m_* and *g_T_* is nonlinear, resulting in inconsistent SNR variations.

## 4. Conclusions

In conclusion, the high-temperature sensing characteristics of standard silica SMF based on polarized and depolarized GAWBS are demonstrated experimentally. A nearly linear relationship between the resonance frequency of GAWBS spectrum and temperature is obtained with a linearly fitted temperature-dependent resonance frequency shift coefficient of 16.74 kHz/°C for *R*_0,4_ mode, and 8.19 kHz/°C for *TR*_2,7_ mode. Structural simplicity and operational advantages confirm the potential of the method for high-temperature sensing in harsh environments. The upper temperature limit of the sensor depends on the melting point of the FUT, higher temperature detection is possible with GAWBS in special high temperature resistant fibers. More importantly, the SNR of the GAWBS characteristic peak is found to increase with increasing temperature, which is attributed to the increased viscose lifetime of acoustic phonons involved in the GAWBS process. The relationship between the gain coefficient and temperature is also analyzed by numerical simulations. The detailed characterization of thermal response of GAWBS in silica SMF to temperature will provide valuable guidance and references for various sensing scenarios with GAWBS in extreme environments, such as nuclear power plants, aerospace, and deep underground wells.

## Figures and Tables

**Table 1:** Calculated and measured resonance frequencies of the polarized and depolarized GAWBS in silica SMF.

Order of Acoustic Mode (*m*)	Resonance Frequencies of Polarized GAWBS		Resonance Frequencies of Depolarized GAWBS	
	Calculation (MHz)	Measurement (MHz)	Calculation (MHz)	Measurement (MHz)
1	30.48	30.34	22.32	22.29
2	81.72	81.29	38.34	39.01
3	130.17	130.10	71.16	70.55
4	178.23	178.21	81.31	80.79
5	226.16	226.13	109.01	108.42
6	270.03	272.31	127.88	128.04
7	321.86	321.97	139.51	140.42
8			170.27	169.12
9			177.80	176.34
10			201.81	201.14

## Data Availability

Not applicable.
